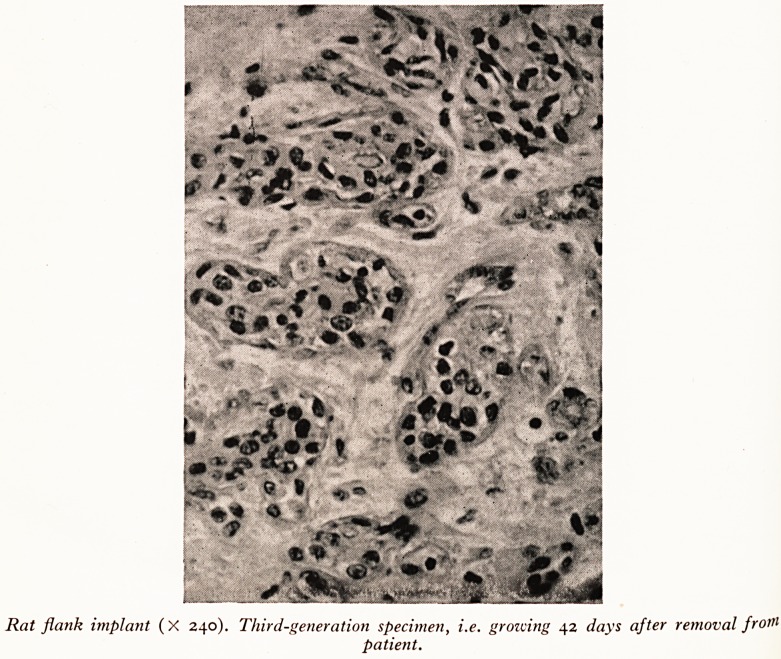# The Growth of Human Tumours in Laboratory Animals

**Published:** 1956-07

**Authors:** G. J. Hadfield

**Affiliations:** Department of Surgery, Bristol Royal Infirmary


### THE GROWTH OF HUMAN TUMOURS IN LABORATORY ANIMALS

### A new approach to the Assay of Anti-Carcinogens

BY

G. J. HADFIELD, M.S., F.R.C.S.
*Department of Surgery, Bristol Royal Infirmary*

Although metastatic breast cancer is at present beyond surgical cure the disease can be palliated by removal of the ovaries and adrenal glands. The first adrenalectomy was performed by Charles Huggins of Chicago in 1951. After operation the secondaries in some of the cases rapidly melt away but this effect is unfortunately only temporary.

One advantage of this type of clinical treatment however is that we have learned indirectly how cancer can be influenced by altering the body tissues in which the tumour grows.

Another line of attack on this problem is that of transplanting human tumours into laboratory animals. This provides a means of studying the effects of certain chemicals, hormones and viruses on human breast cancer outside the patient and gives a laboratory testing-ground for them before trial on patients.

This paper describes briefly how human tumours can be grown in rats and hamsters for this purpose. The method is not new for in 1913 Carrel and Burrows working in this country showed that human tumours could be grown when implanted into animals. More recently Dr. H. S. N. Greene of Yale University and Dr. H. Toolan of the Memorial Center in New York have revived and developed this method. They have found that transplants will grow when inoculated into the brain, anterior chamber of eye, the subcutaneous tissues of the rat or the cheek pouch of the hamster.

The technique of the cheek-pouch implant in the hamster is very simple. The animal is anaesthetized and a piece of tumour the size of a large pin's head is introduced into the cheek pouch through a small puncture wound. Plate VI (*a*) shows the tumour growth from an implant after eighteen days.

Subcutaneous implants are carried out in rats. A fine suspension of minced tumour in a buffered solution is injected into the subcutaneous tissues of one flank through a wide bore needle and syringe. Plate VI (*b*) shows one of the implants growing in rat.

For the tumour to grow in the animal it has to obtain a new supporting stoma and blood supply locally from the rat's tissues. At this stage the unprotected cancer cells of the implant are highly vulnerable to the host reaction. For the implant to survive this the animal's tissue reaction has to be temporarily modified. This can be effected by giving small doses of cortisone, X-rays or a combination of the two.

Human cancer grown by these means in animals can, once it is established, be used as a method of testing anti-cancer substances. Subcutaneous or submucous tumours are easily accessible to direct palpation to assess changes in size. Direct measurement of changes can also be made with a pair of calipers.

One criticism of this method has been that these human tumours growing in animals lose their human characters and become an animal tumour. This has been nullified by some recent work carried out at the Memorial Center in New York. A specimen of highly anaplastic cancer of the cervix was obtained from an inoperable case. This was grown in rats and hamsters on serial transplantation by the methods previously described. Later with the patient's co-operation and wish it was re-transplanted beneath the skin of the forearm. Two weeks later the transplant was excised and by that time it was again growing well. This seems to abolish the criticism that these human tumours grown in animals lose their human character.

PLATE VI

(*a*) *Human breast cancer implants in hamster cheek pouches,* 18 *days after inoculation.*

(*b*) *Human breast cancer implant injected below skin of rat flank,* 14 *days growth. (Note seedling growths along needle track and that the tumour is obtaining a blood supply from the host.)*

*Original tumour* (× 240).

*Rat flank implant* (× 240). *Third-generation specimen, i.e. growing* 42 *days after removal from patient.*

This technique also provides an easy method of observing the behaviour of cancer cells in normal and cancerous animals. It provides a method of studying the growth and spread of cancer and the question of resistance and immunity to the tumour process.

Plate VII is a histological specimen of human breast cancer growing in the rat's flank. The original, shown with it for comparison came from a primary cancer of the breast removed by radical mastectomy.

(This work was carried out during the tenure of a British Empire Cancer Campaign Fellowship at the Memorial Center, New York.)

## Figures and Tables

**(a) f1:**
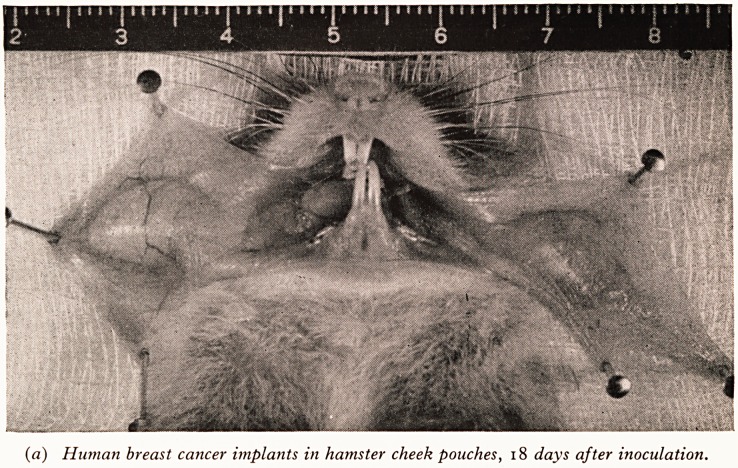


**(b) f2:**
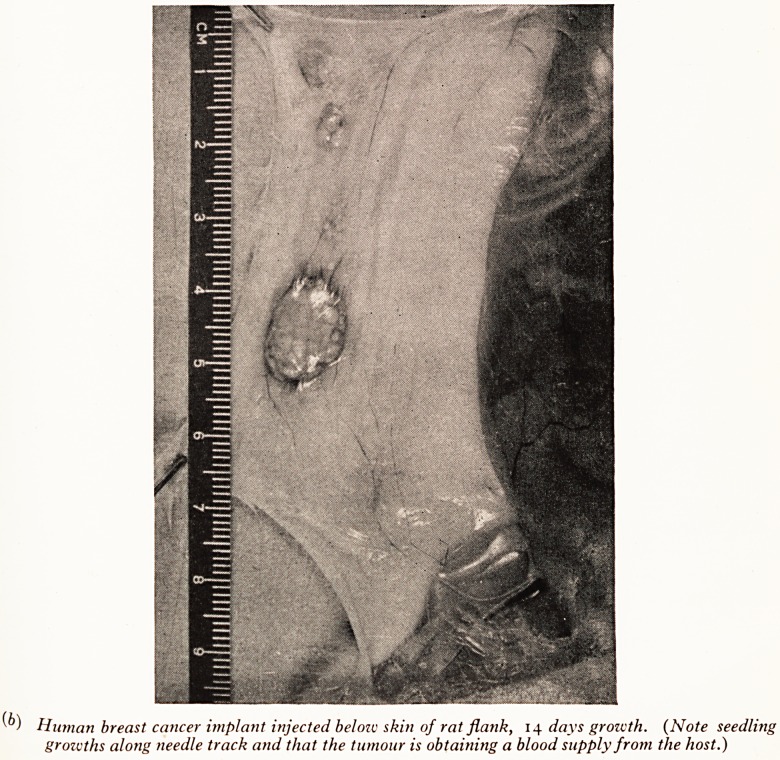


**Figure f3:**
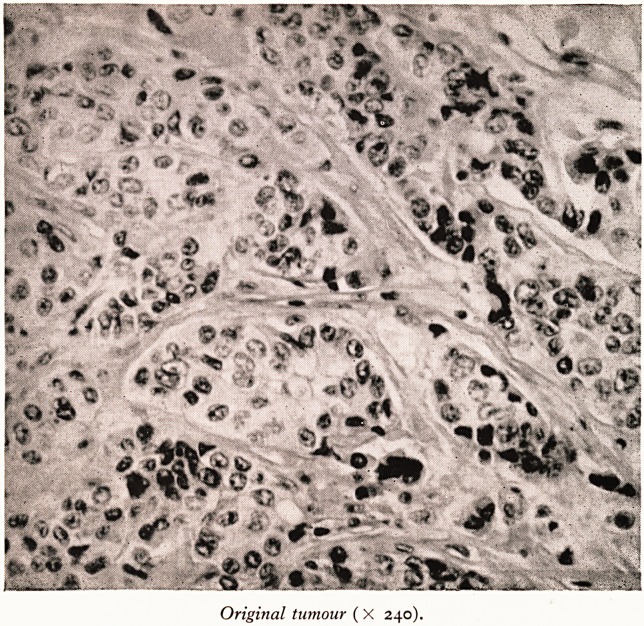


**Figure f4:**